# A transcriptome study of p53-pathway related prognostic gene signature set in bladder cancer

**DOI:** 10.1016/j.heliyon.2023.e21058

**Published:** 2023-10-14

**Authors:** Safayat Mahmud Khan, Tonmoy Das, Sajib Chakraborty, Abdul Matin Anamur Rashid Choudhury, Howlader Fazlul Karim, Munshi Akid Mostofa, Hasib Uddin Ahmed, Md Akmal Hossain, Florence Le Calvez-Kelm, Md Ismail Hosen, Hossain Uddin Shekhar

**Affiliations:** a Clinical Biochemistry and Translational Medicine Laboratory, Department of Biochemistry and Molecular Biology, University of Dhaka, Dhaka, Bangladesh; b Systems Cell-Signalling Laboratory, Department of Biochemistry and Molecular Biology, University of Dhaka, Dhaka, Bangladesh; c Department. Uro-Oncology, National Institute of Cancer Research Hospital, Bangladesh; d Genomic Epidemiology Branch, International Agency for Research on Cancer (IARC), 69372, Lyon, France

**Keywords:** NMF, BLCA, p53, Biomarker, Enrichment, TCGA, Signature-set, Nomogram, DLX1, DSC1

## Abstract

p53 pathway is important in tumorigenesis. However, no study has been performed to specifically investigate the role of p53 pathway genes in bladder cancer (BLCA). In this study, transcriptomics data of muscle invasive bladder cancer patients (n = 411) from The Cancer Genome Atlas (TCGA) were investigated. Using the hallmark p53 pathway gene set, the Non-Negative Matrix factorization (NMF) analysis identified two subtypes (C1 and C2). Clinical, survival, and immunological analysis were done to validate distinct characteristics of the subtypes. Pathway enrichment analysis showed the subtype C1 with poor prognosis having enrichment in genes of the immunity related pathways, where C2 subtype with better prognosis being enriched in genes of the steroid synthesis and drug metabolism pathways. A signature gene set consisting of *MDGA2, GNLY, GGT2, UGT2B4, DLX1, and DSC1* was created followed by a risk model. Their expressions were analyzed in RNA extracted from the blood and matched tumor tissues of BLCA patients (n = 10). *DSC1* had significant difference of expression (p = 0.005) between the blood and tumor tissues in our BLCA samples. Contrary to the usual normal bladder tissue to blood ratio, *DLX1* expression was lower (p = 0.02734) in tumor tissues than in blood. Being the first research of p53 pathway related signature gene set in bladder cancer, this study potentially has a substantial impact on the development of biomarkers for BLCA.

## 1 Introduction

Bladder Cancer (BLCA) is the tenth most common cancer globally, the sixth most common in males, with a tenfold increased risk of incidence compared to women [1]. Bladder cancer cases are classified as urothelial non-muscle invasive bladder (NMIBC) cancer when a tumor is confined to the mucosa, and the muscle-invasive bladder cancer (MIBC), with invasion of deeper layers [[2], [3], [4]]. Risk factors include smoking (responsible for more than 50 % of BLCA in developed regions), occupational and environmental exposures [5,6].

Despite years of study and advancements in diagnostic and monitoring methods, endoscopic and imaging techniques, the detection of BLCA largely rely on cystoscopy, sometimes combined with urine cytology [7]. Screening in high-risk groups, such as heavy smokers and personnel exposed to chemicals associated with a higher risk of BLCA is not recommended by international guidelines, mainly because of the cost and invasiveness of cystoscopy. However, subjects with persistent microhematuria, also at high risk of BLCA are advised to follow further cystoscopy and imaging examinations based on a risk-stratified model [[7], [8], [9]]. Several studies have been conducted for developing molecular biomarkers for BLCA, but the detection of low-grade BLCA has been proved inconclusive in most cases [10,11]. Gene expression profiles are often used as prognostic or predictive biomarkers. Prognostic signatures derived from transcriptome-wide studies might aid in stratifying patients into different risk groups, thereby facilitating personalized treatment [12,13].

Prognostic Gene Signature sets study is still relatively new in BLCA patients [3]. Mostly, key markers and actionable targets related to the Lund subtyping classification have been identified, mainly related to the immune checkpoints, *FGFR3, EGFR,* and *ERBB2* genes [[14], [15], [16], [17]]. Recently, investigating the specific molecular pathway-based gene sets along with predictive modeling has been seen as an innovative approach for the identification of prognostic gene signature sets in the specified pathway(s) [13,18]. On this front, the transcriptome-wide analysis of the RNA-seq data has ushered in a new age of analysis-based research, based on a collective study of a huge dataset of genes and samples rather than one single point/target gene.

The p53 pathway is a network of genes and their products that respond to internal and external stress signals that regulate DNA replication, chromosomal separation, and cell division, involving positive and negative feedback loops linking the p53 pathway to other signaling channels in the cell [19]. The p53 gene and pathways are altered in the majority of aggressive, invasive bladder carcinomas [20]. Numerous studies have demonstrated alterations in p53 expression in bladder tumors, most frequently in connection with advanced neoplasia. These alterations are considered to be predictive of survival in a variety of malignancies and may also be predictive of treatment response [21]. A study of 243 BLCA patients who had radical cystectomy found a substantial link between p53 nuclear accumulation and reduced recurrence-free, and overall survival across all stages of the disease [22].

The aim of our study was to investigate the transcriptome data from the TCGA-BLCA dataset [23], and construct a prognostic signature gene set using different bioinformatic approaches. Further, we studied expression of the selected genes in the gene set in a cohort of BLCA patients.

## 2 Materials and methods

### 2.1 Study design

**Fig. 1:**
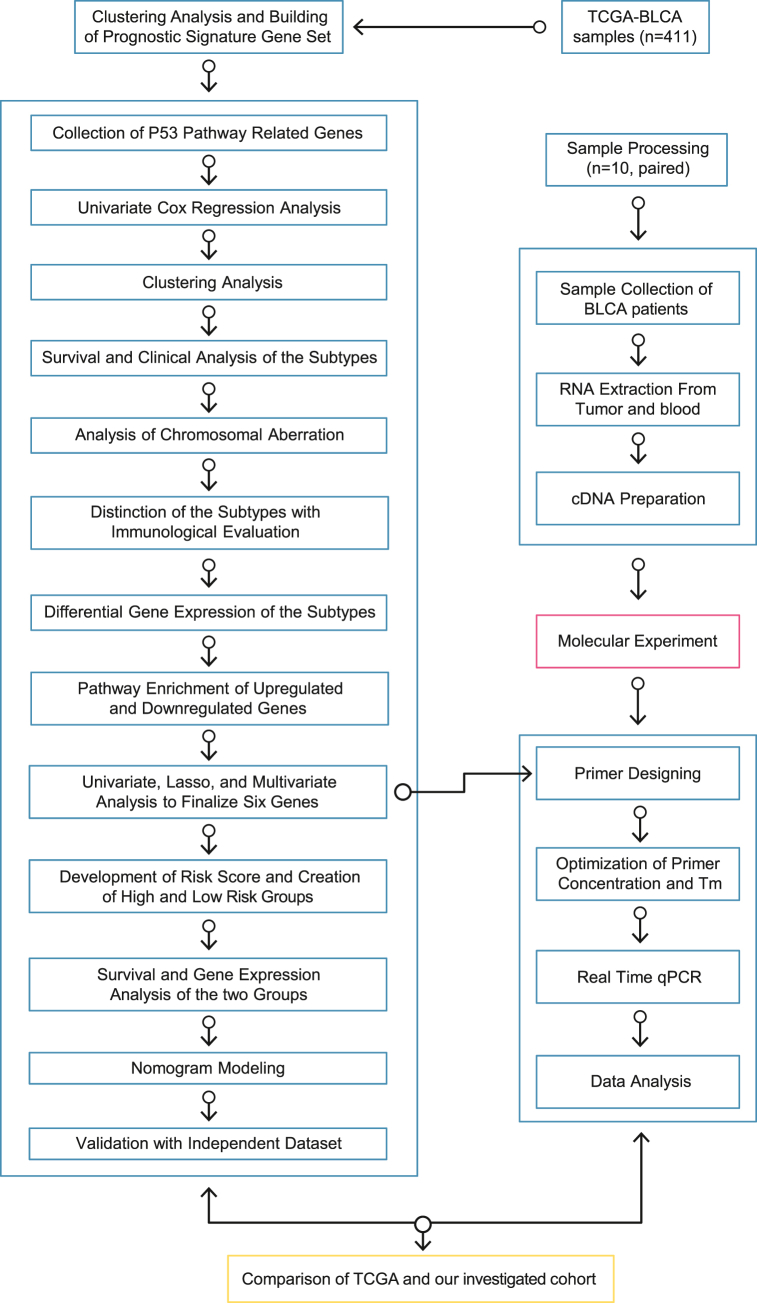
Diagram detailing the research methodology of the study.

This study was designed to investigate the TCGA dataset from MIBC patients (n = 411) to predict a signature gene set on the basis of subtyping with p53-pathway related genes [23]. Overall, this study had two phases. The first one was the analysis of TCGA expression data to create a prognostic signature gene set based on the p53-pathway. The next one was to investigate the expression of the genes in the constructed gene set in a set of independent bladder cancer patients (n = 10). Data from each study were then compared to evaluate whether the gene set derived from the TCGA data could stratify BLCA patients into different risk groups. The research methodology for the whole study is given in Fig. 1.

### 2.2 Collection of data

The “HALLMARK_P53_PATHWAY” gene set was collected from The Molecular Signatures Database v7.5.1 (MSigDB; https://www.gsea-msigdb.org/gsea/msigdb). In total, the gene set included 200 genes [24]. By utilizing the TCGA-BLCA cohort, this study obtained the mRNA expression as well as corresponding clinical information data (survival status) from the TCGA pan-cancer atlas (TCGA, http://cancergenome.nih.gov/). The additional clinical information was retrieved from the cBioPortal which included-cancer metastasis stage, neoplasm disease stage, neoplasm status, and survival-follow up (Supplementary Table 1) [25]. Data from a total of 411 Muscle Invasive BLCA samples from the TCGA were utilized in this study.

### 2.3 Classification of two p53 pathway-related molecular subtypes based on clustering analysis

A univariate cox regression survival analysis was carried out based on the p53 hallmark pathway genes to assess the TCGA-BLCA cohort's overall survival (OS) status. The genes with a p-value of <0.05 were taken for further evaluation. Two R packages ‘survival’ and ‘survminer’ were used for the analysis. The TCGA-BLCA dataset was then subjected to unsupervised non-negative matrix factorization (NMF) clustering using the R package ‘NMF’ using the significant genes found from the univariate cox regression analysis [26]. The ideal number of clusters was determined by determining the k-value at which the cophenetic correlation coefficient began to fall. The “Brunet” method was applied to do the test with a rank of 2:6 and nrun = 50. The principal components analysis (PCA) of the transcriptome expression profiles of the p53 pathway-related genes was carried out in order to assess the classification performance. Then, the Fviz_cluster method was applied to draw the PCA plot by k-means clustering with k = 2. The ‘survival’ package in R was used to create Kaplan-Meier overall survival (OS) curves, which were then tested using the log-rank test.

### 2.4 Analysis of the amplification and deletion of chromosomes

The amplification and deletion of chromosomes were exhaustively explored. The Genomic Identification of Significant Targets in Cancer (GISTIC) v2.0 was used for this purpose (https://software.broadinstitute.org/cancer/cga/gistic). The Copy number variation (amplification and deletion) plots for the respective subtypes were generated in a genome-wide manner after inputting the two-sample subtype GISTIC scores [27].

### 2.5 Evaluation of stromal and immune cell composition and assessment of tumor purity of the samples

The ESTIMATE algorithm was used to determine the number of infiltrating stromal and immune cells in the TCGA-BLCA samples [28]. Besides, the purity of each tumor sample was determined by applying four different methods including- ESTIMATE, which is based on gene expression profiles for 141 immune and 141 stromal genes; ABSOLUTE, which is based on somatic copy-number data; LUMP (leukocytes unmethylation for purity), which is based on an average of 44 non-methylated immune-specific CpG sites; and IHC, which is based on image analysis of haematoxylin and eosin stain slides generated by the Nationwide Children's Hospital Bio. The final method to be used was consensus measurement of purity estimations (CPE) which is the median purity level after normalization from all other methods to give them an equal score. The purity data for the mentioned algorithms were extracted from a previous study [29].

### 2.6 Deconvolution of TCGA-BLCA bulk RNA-seq data

The CIBERSORTx algorithm was used to determine the infiltration levels of 22 immune cell types in each of the BLCA samples from TCGA data [30]. Briefly, the default 22 functionally defined human immune subsets (LM22) file from the CIBERSORTx tool was used signature matrix for this study. The normalized gene expression matrix of the TCGA data was then used as an input mixture file. This value of the gene expression was obtained from the UCSC Xena server (https://xena.ucsc.edu/) [31] The cell type fraction values obtained for each sample from the CIBERSORTx analysis were subjected to statistical analysis where the Wilcoxon rank-sum test was used to determine whether there were variations in immune infiltration levels between the respective subtypes.

### 2.7 Evaluation of chemokines and immune checkpoint genes between subtypes

Significant chemokines (*CXCL1*, *CXCL2*, *CXCL5*, *CXCL10*, *CXCL11*, and *CXCL13*) and immune checkpoint-related genes (*CD274*, *CTLA4*, *IDO1*, *LAG3*, *PDCD1*, and *PDCD1LG2*) were retrieved by literature mining [[32], [33], [34], [35], [36], [37]]. These genes were found highly associated with BLCA. The expression levels of these genes between the two subtypes were analyzed further by Wilcoxon rank-sum test.

### 2.8 Differential expression and functional enrichment analysis

The ‘DEseq2’ package from Bioconductor was implemented to filter differentially expressed genes (DEGs) between the two molecular subtypes, with a cutoff of FDR<0.05 and log2 (Fold Change) > 1. HT-seq count data was used as input, and the count data was normalized. The significant genes were subtyped as C1 upregulated, C2 upregulated, and neutral. Functional enrichment analysis was done for the regulated genes for the two subtypes through the Gene Set Enrichment Analysis (GSEA). Using the Gene Ontology (GO) and Kyoto Encyclopedia of Genes and Genomes (KEGG) gene-sets, the enrichment analysis was performed to predict their underlying functions. Gene-sets with an FDR value of less than 0.05 was denoted significant.

### 2.9 Establishment of a signature gene set based on DEGs in two molecular subtypes

A univariate cox proportional hazards regression analysis was carried out on all of the DEGs and the survival data. The “coxph” function from the ‘survival’ package of R, with a log rank of p < 0.05 was used as the criterion for survival analysis. Additional gene range narrowing was necessary to construct a prognostic model with a high accuracy rate, and the least absolute shrinkage and selection operator (Lasso) technique was used to estimate compression. It established a penalty function in order to build a more exact model, which resulted in the compression of certain coefficients while setting others to zero [38]. The lambda value was taken as the lambda minimum to get the least mean standard error.

### 2.10 Construction of a risk formula and risk model

Multivariate regression analysis was performed for the significant genes found from the previously mentioned Lasso model. Using the regression coefficient and RNA-seq expression log2 (fpkm+1), a risk formula was generated. The formula is:

RiskScore=Coefficient×(ExpressionofDLX1)+Coefficient×(ExpressionofDSC1)+Coefficient×(ExpressionofMDGA2)+Coefficient×(ExpressionofUGT2B4)+Coefficient×(ExpressionofGNLY)+Coefficient×(ExpressionofGGT2)

The TCGA-BLCA patients were classified into high- and low-risk groups based on the risk scores. The survival package was used to produce Kaplan-Meir curves to compare the two groups' overall survival [39]. Receiving operating characteristic curves (ROCs) for one-, three-, and five-years OS were calculated using the ‘timeROC’ package to examine the prediction potential of the risk score. The timeROC package was used to calculate the ROCs [40]. A receiver operating characteristic (ROC) curve is created by comparing the true positive rate (TPR) vs false positive rate (FPR). The true positive rate is the fraction of positive observations that were properly expected to be positive (TP/(TP + FN)). Similarly, the false positive rate is the fraction of observations mistakenly projected as positive relative to all negative observations (FP/(TN + FP)). It is a useful tool for assessing the effectiveness of a potential marker based on the real illness state of people at certain time points. The expression pattern of the six gene signature set was evaluated using an unsupervised clustering method with the R package ‘pheatmap’.

### 2.11 Construction of a nomogram model

The ‘hdnom’ package in R was used to create a nomogram for predicting overall survival that took into account clinical parameters and the risk score. The scores assigned to variables were determined by the correlation coefficients between them. To get a total score for each patient, the sum of all the individual scores for each variable was computed. The probability of each patient's outcome was then estimated using the conversion function. Calibration plots were used to assess the prediction efficacy of the nomogram [41]. Lasso model was chosen for fitting the nomogram with nfold of 5. For the calibration, a bootstrapping method was applied with a penalty factor = adapen, and boot. times = 10.

### 2.12 Validation of the robustness of the six gene signature in the independent dataset

External validation of our gene signature set was performed using the GEO dataset GSE13507. BLCA patients (n = 165) were split into high- and low-risk groups according to the threshold value for risk scores calculated using the signature gene set's risk score. Kaplan-Meir curves were generated [39]and the timeROC package was used to calculate the ROCs [40].

### 2.13 General statistical analysis

The statistical analysis was done by R language v4.0.2 (https://www.r-project.org/) with a p-value <0.05 (two-tailed) representing significant significance. Normality distribution test was done by Shapiro Wilk test and comparison between the groups were done by the Wilcoxon rank-sum test. The survival analysis was done by the log rank test. The chi-squared test was used to assess the differences between the groups of category variables.

### 2.14 RNA isolation and quantitative real-time polymerase chain reaction (qRT-PCR)

Ten pairs of matched blood samples and bladder tumor tissues were collected from BLCA patients being from June 2021, to February 2022. By using PureLink® RNA Mini Kit (Thermo Fisher Scientific, Waltham, Massachusetts, United States) manual, Catalog numbers: 12183018A -total RNA was extracted from whole blood, and Tumor tissue. ProtoScript® II First Strand cDNA Synthesis Kit (New England Biolab, Ipswich, Massachusetts, United States) was used to synthesize cDNA from total RNA. PowerUp™ SYBR™ Green Master Mix (Thermo Fisher Scientific, Waltham, Massachusetts, United States) was used to do real time quantitative PCR (qRT-PCR), analysis was done using qTOWER³G (analytikjena). The genes and primers are given in Supplementary Table 2.

Actin Beta (*ACTB*) gene was used as an internal control (housekeeping gene). Four target genes- *MDGA2*, *GNLY*, *DLX1*, *DSC1* and the *ACTB* housekeeping gene were amplified and their gene expressions were measured with fold-changes calculation with the 2^-ΔΔ Ct method (Livak method) [42]. Triplicates of each sample were done for qPCR analysis. For each plate, three target genes and one housekeeping gene for three samples were plated and data were analyzed. After the experiment, housekeeping gene ACTB's value was used to normalize the target genes' expression and finally 26-ΔΔ Ct value was gained as expression value to analyze the data.

### 2.15 Generation of a risk model followed by subgrouping

A risk formula was created with the gene expression values of the three genes- *DSC1, DLX1, GNLY* in our cohort. With the formula, risk scores were generated for each of the ten tumor samples and two groups (high-risk and low-risk), each containing 5 samples. Further analysis will be done when the tumor recurrence and survival data will be available.

RiskScore=Coefficient×(ExpressionofDLX1)+Coefficient×(ExpressionofDSC1)+Coefficient×(ExpressionofGNLY)

## 3 Results

### 3.1 Non-negative matrix factorization of p53 pathway genes stratifies the BLCA patients into distinct molecular subtypes

There is evidence that p53 pathway is involved in stress responses in cells, including DNA repair, cell-cycle arrest, senescence, and the most significant apoptosis inducing factor [43]. The “HALLMARK P53 PATHWAY” gene set from the MsigDB database yielded 200 p53 pathway genes. The TCGA-BLCA cohort included 411 patients with MIBC whose expression data for these 200 hallmark-associated genes were extracted and subjected to a univariate cox regression analysis. A total of 45 genes correlating with BLCA prognosis (p < 0.05) were identified (Supplementary Table 3).

**Fig. 2:**
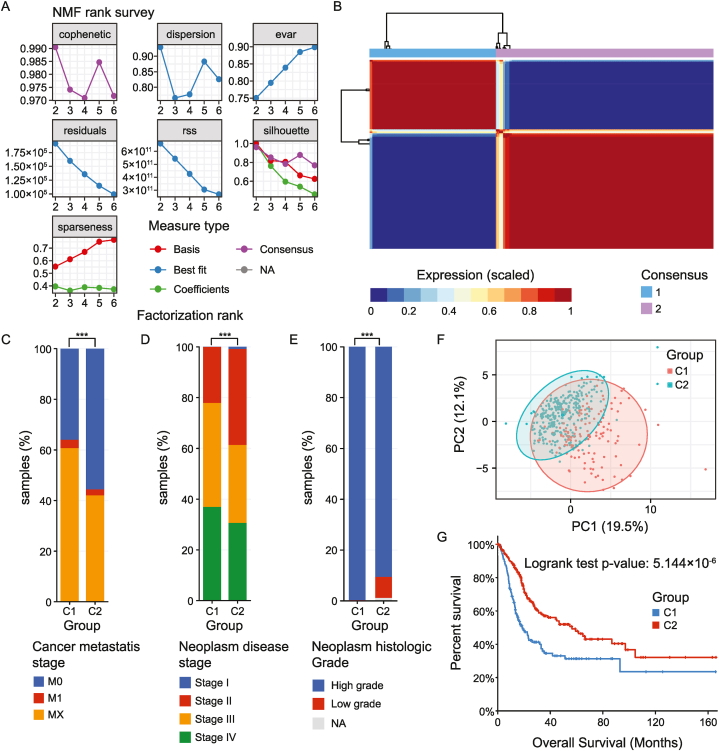
In the TCGA-BLCA dataset, NMF reveals two unique p53 pathway-related molecular subtypes for BLCA. **(A)** Factorization rank for k = 2–6. **(B)** The consensus matrix's heat map when k = 2. The range of values is 0–1. The columns and rows are sorted using hierarchical clustering according to the average link's Euclidean distance. With k = 2, or two clusters, the samples are arranged symmetrically, indicating a perfect clustering number. **(C)** Comparison of Cancer metastasis stage, **(D)** Neoplasm disease stage, **(E)** Neoplasm histologic grade between the two subtypes. All the data showed statistical significance between the subtypes (*P = <0.5, **P= <0.05, ***P= <0.005). **(F)** PCA analysis of the samples. K-means was done first for the clustering with n = 25. **(G)** Kaplan-Meier overall survival curves for the two clusters of TCGA-BLCA dataset. The log-rank test was used to determine the significance level between the two groups.

To identify the BLCA molecular subtypes based on the p53 pathway genes, we performed the NMF analysis applying cophenetic, dispersion, and silhouette features, which resulted in the formation of two different subtypes. Beginning with the rank, k = 2, the cophenetic correlation coefficient began to drop (Fig. 2A). When k = 2, the heatmap intuitively displayed the consensus matrix with two distinct clusters (Fig. 2B). The consensus matrix of the samples divided into three clusters did not generate an ideal heatmap (Supplementary Fig. 1). As a result, BLCA samples were classified into two molecular subtypes – C1 (n = 155) and C2 (n = 256). Clinical assessment was done for these two groups of BLCA patients. The comparison was done on the basis of Cancer metastasis stage, Neoplasm disease stage, and Neoplasm histologic grade between two subtypes (Fig. 2C–E). For the cancer metastasis, stage-C1 showed a higher percentage of MX samples which means spread of metastasis can't be measured. On the other hand, C2 showed a higher percentage sample of M0 which means cancer has not spread to another organ. There was a higher percentage of stage 4 and tumor diagnosed patients in C1 subtypes. The p-values are 0.000653 (Cancer metastasis stage), 0.005263 (Neoplasm disease stage), 4.256 × 10–4 (Neoplasm histologic grade) between two subtypes. PCA supported the classiﬁcation into two subtypes. Also, k-means was calculated for the sample subtypes and clustering showed samples with variance prediction of 12.1 % and 19.5 % (Fig. 2F). The significant prognostic difference between C1 and C2 was studied in the TCGA-BLCA cohort with Kaplan-Meier survival analysis. The C1 group showed a poor overall survival (OS) outcome in comparison to the C2 group (Fig. 2G). Log rank-Test value was done to find out the p-value (5.144 × 10^−6^) between these two groups. The disease free, progression free, disease specific survival analysis plots are given in Supplementary Fig. 2 and the survival plots of the samples divided into three clusters are given in Supplementary Fig. 3.

### 3.2 Copy number variation analysis shows genomic heterogeneity in distinct BLCA molecular subtypes

To understand the genomic heterogeneity of the BLCA subtypes, we performed the copy number variation (CNV) analysis on C1 and C2 samples. The frequencies of copy number variation (CNV) in BLCA samples from two subtypes were observed (Supplementary Fig. 2). The most frequently amplified chromosomes were chromosome 1 and chromosome 6 in both the C1 and C2 subtypes. Meanwhile, the most common deletion sites were found on chromosome 9. C1 had a lower frequency of amplification and deletion than C2 (p-value = <2.2 × 10^−16^) (Supplementary Fig. 4). A chi square test was done between the two clusters for a mutation vs no variant category for the gene *TP53* which was insignificant (p value = 0.450034) (Supplementary Table 4). Also, CNV analysis was further extended to observe deletion and amplification sites of the p53 pathway target genes. Some of the deleted and amplified genes were from the finalized 45 genes of our NMF analysis. Both C1 and C2 had *TP63* and *ZMAT3* amplified which were two of the genes from the 45 finalized genes. There were dissimilarities in deletion of genes for the two clusters. Out of the 45 genes, *EPS8L2, FBXW7, HSPA4L, SLC7A11*, and *ST14* were common deletions in the two clusters. For C1 cluster *APP, INHBB, RAB40C, RAD9A, SLC3A2, TRAF4, VAMP8* had deletions whereas C2 had deletions in *ABHD4, ANKRA2, EPHX1, F2R, FOXO3, FUCA1, IP6K2, PLK2, PTPN14, TNNI1* genes (Supplementary Table 5). None of the deletion sites from the final set of genes were in chromosome 9.

### 3.3 Disparate immune infiltration defines the distinct BLCA subtypes

**Fig. 3:**
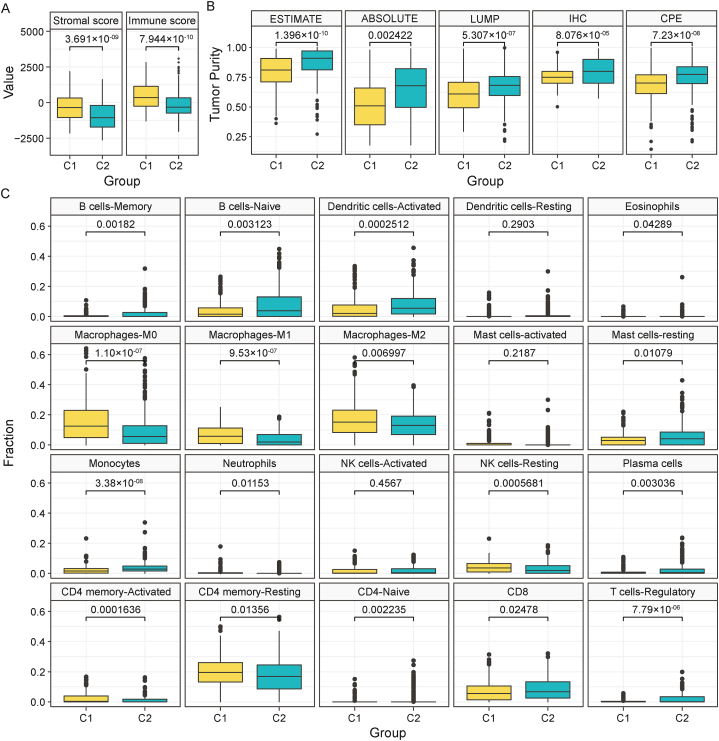
**(A)** Stromal score and Immune score comparison between C1 and C2 subtypes. **(B)** Tumor purity scores between the subtypes C1 and C2 using five different algorithms-ESTIMATE, ABSOLUTE, LUMP (leukocytes unmethylation for purity, IHC (image analysis of haematoxylin and eosin stain slides), CPE (median purity level after normalization from other four methods to give them an equal score). **(C)** CIBERSORTx analysis illustrating cell fraction value for multiple tumor-infiltrating immune cell types.

The contribution of stromal and immune scores in the defined C1 and C2 groups of BLCA may identify the role of tumor microenvironment on tumorigenesis and homeostasis [28]. The stromal and immunological score was computed by using data from the ESTIMATE algorithm which focuses on the stromal and immune cells that comprise the majority of non-tumor components in tumor samples. Comparisons between C1 and C2 groups showed statistical significance with a p-value of 3.691 × 10^−09^ (Stromal Score) and 7.944 × 10^−10^ (Immune Score). The stromal and immune scores for all TCGA-BLCA samples are given in Supplementary Table 6. Higher levels of the stromal score and immune score observed in the C1 group indicate a worse prognosis for the C1 subtype validating the previous survival analysis of this study (Fig. 3A; Fig. 2G).

Next, tumor purity levels were also determined using 5 methods ESTIMATE, ABSOLUTE, LUMP, IHC, and CPE (Fig. 3B). The analysis revealed a distinct difference in the tumor purity score between the subtypes where C1 clearly showed significantly lower tumor purity, which has been previously associated with poor prognosis. The TCGA-BLCA sample data for these 5 algorithms are given in Supplementary Table 7. Besides, the immune features can be further investigated by cell-type-specific gene expression profiles of CIBERSORTx. To learn more about the relationships between the subtypes and 22 tumor-infiltrating immune cells (TIICs), CIBERSORTx based deconvolution analysis was used. CIBERSORTx method quantified the 22 TIIC profiles necessary for respected samples of this study. The outcomes were compared between the subtypes C1 and C2. The immune cell types showed a distinct difference in abundance between the two subtypes (Fig. 3C; Supplementary Table 6). Briefly, memory B-cells, naïve B-cells, activated dendritic cells, eosinophils, resting mast cells, monocytes, plasma cells, naïve CD4-T cells, CD8-Tcells and T-regulatory cells showed greater abundance of C2 subtype in their composition in comparison to the C1 subtype. On the other hand, macrophages (M0, M1, and M2), resting NK cells, activated CD4-memory T-cells, and resting CD4-memory T-cells exhibited greater fraction of C1 subtype in comparison to the C2 subtype. The rest of the cell types didn't display any significant difference between the two groups (Fig. 3C; Supplementary Table 8).

### 3.4 Differential expression of chemokines and immune checkpoint genes in BLCA molecular subtypes

**Fig. 4:**
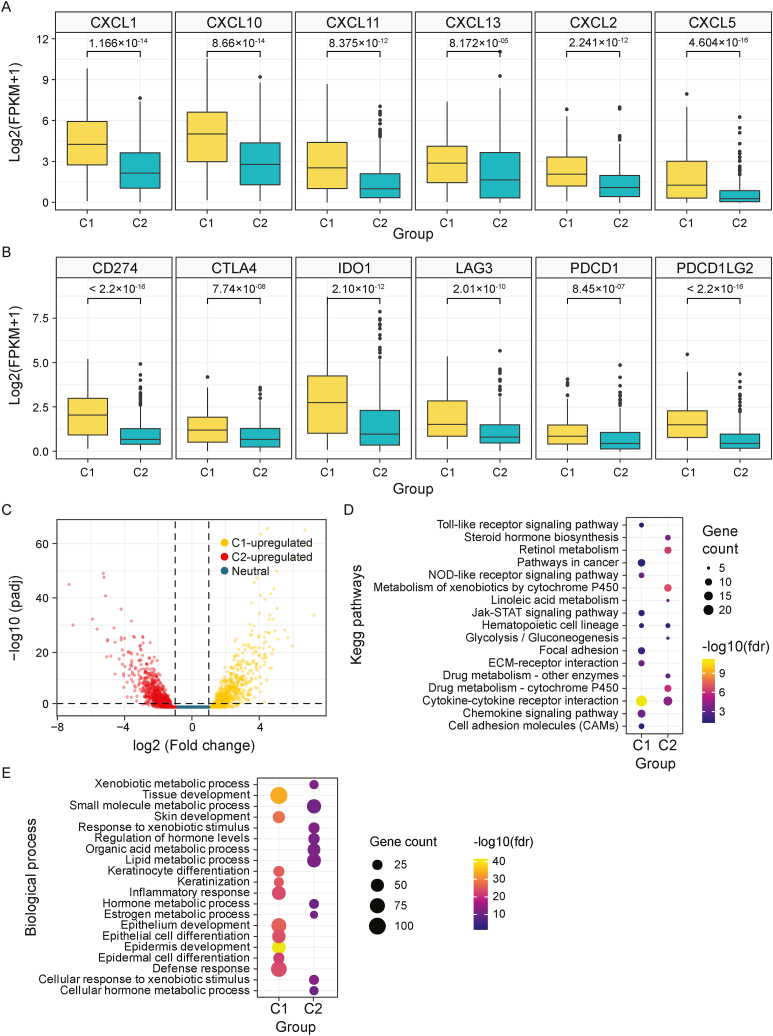
**(A)** Comparative analysis of chemokine expression in C1 and C2 subtypes. **(B)** Gene expression score of immune check point gene- *CD274, CTLA4, IDO1, LAG3, PDCD1, PDCD1LG2* between the two subtypes C1 and C2. **(C)** DEGs visualized between the 2 subtypes. Yellow color dots represent upregulated genes in C1 compared to C2, while red color dots indicate upregulated genes in C2 compared to C1. In the middle blue region defines neutral genes. **(D**–**E)** Functional enrichment analysis of KEGG pathway **(D)** and GO based biological process pathway the between C1 and C2 upregulated genes.

Chemokines may directly control tumor development by stimulating cancer cell proliferation and inhibiting their apoptosis [33,34,44]. The expression levels of *CXC* chemokines were measured between the two subtypes. Only the chemokines related to bladder cancer were analyzed. C1 subtype showed higher expression *in CXCL1, CXCL2, CXCL5, CXCL10, CXCL11, and CXCL13* (Fig. 4A). Also, critical immune checkpoint genes such as *LAG3, PDCD1LG2, CD274, IDO1, PDCD1*, and *CTLA4* were evaluated because these genes are activated when T cells identify and attach to associated proteins on other cells, such as certain tumor cells (Fig. 4B). The C1 subtype showed significantly greater expression all of these genes compared to the C2 subtype (Fig. 4A and B).

### 3.5 Distinct BLCA molecular subtypes harbor heterogeneous transcriptomic landscapes

The differentially expressed genes between subtypes C1 and C2 were selected based on the following criteria: FDR <0.05 and |log2 FC| ≥1. In total, 3757 DEGs were found using the Bioconductor package DEseq2 (Supplementary Table 9). Among these, 1522 genes were upregulated and 2235 genes were downregulated in C1 vs C2. (Fig. 4C). Functional enrichment analysis was done with the KEGG pathway (Fig. 4D) and Gene ontology (GO) terms pathway (Fig. 4E; Supplementary Fig. 5). While C1 subtype upregulated genes were enriched in Immunity related pathways, C2 upregulated genes were enriched in especially steroid hormone synthesis and drug metabolism pathways. Besides, for the C1 upregulated genes, the Cytokine-Cytokine receptor pathway, Toll-like receptor signaling pathway, pathways in cancer, etc. Had greater enrichment scores and significance. For the C2 upregulated genes, major enriched pathways are related to cytochrome P450 related pathways, hormone, lipid synthesis, etc. related pathways. (Fig. 4D and E, Supplementary Fig. 5).

### 3.6 Stratifying BLCA patients into risk groups based on the multivariate analysis of the p53 associated genes

**Fig. 5:**
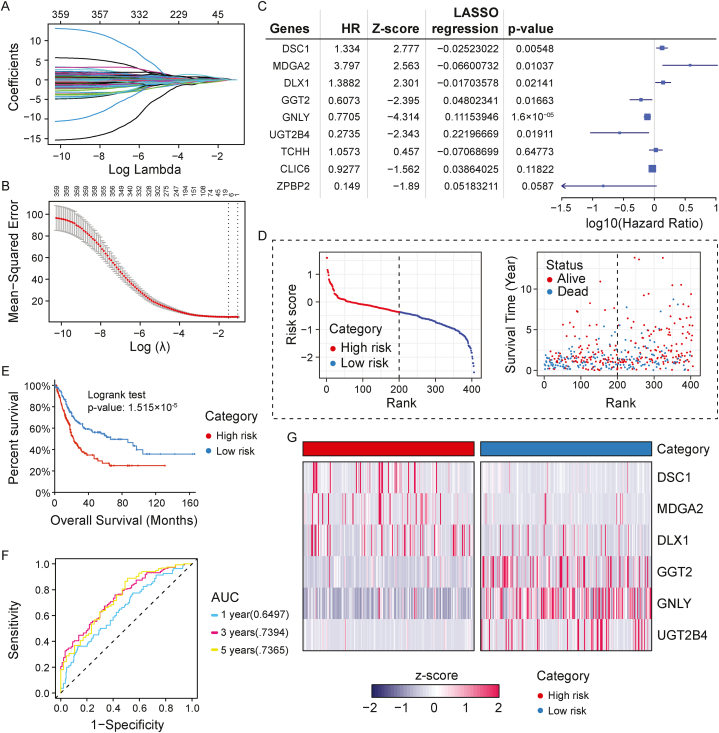
**(A)** LASSO model of DEGs with survival time (year) and **(B)** minimum lambda value (0.2196665). The mean squared is least at the log lambda value which gives an output of nine significant genes. **(C)** Multivariate analysis results of the nine significant genes with LASSO score. **(D)** Illustration of risk score and survival time status along with the rank of samples High-risk area is denser with dead patients compared to low-risk patients. High-risk patients have a comparatively lower survival time (p-value = 0.016). **(E)** Kaplan-Meier overall survival plot for high-risk and low-risk groups based on the risk scores of six gene signature set. **(F)** ROC curve illustrating the prediction potential of the risk model, AUC value showed good results indicating the insignificant False positive rate of the analysis signifying the performance of the risk score for prediction of OS for 1 year, 3 years, and 5 years. **(G)** Heatmap of the six gene signature set in high-risk and low-risk groups. The gene expression data is scaled by the z-score method.

In total, 1112 prognosis-related DEGs with FDR <0.05 between subtypes were identified for BLCA. Performing univariate survival analysis with these genes, resulting in 360 significant genes (p-value <0.05) (Supplementary Table 9). Under LASSO Cox regression analysis of these genes, 9 genes were given a value without zero after applying the minimum lambda value (Fig. 5A-B-C).

Multivariate analysis showed significant results for six genes (Fig. 5C). The six-gene signature was constructed that is composed of *MDGA2, DLX1, DSC1, UGT2B4, GNLY,* and *GGT2* genes*.* The genes *DSC1, DLX1,* and *MDGA2* had an HR value > 1 meaning they can be predicted as the hazardous genes for BLCA, while *GGT2, GNLY*, and *UGT2B4* genes had an HR value < 1 predicting their protective role for survival. Each sample's risk score was determined by a risk formula based on the regression coefficient and gene expression value previously mentioned, and all BLCA patients were divided into high- and low-risk groups based on the risk score after dividing the samples in half (Fig. 5D; Supplementary Table 10). The higher the risk score, the higher the percentage of patients that died. The chi-square test value of risk category against survival status was 5.7487 and p-value was 0.016 showing clear distinct survival pattern between the high- and low-risk groups.

### 3.7 Poor survival outcome of the risk groups

Survival analysis displayed significant differences between high and low risk groups (Fig. 5E). Receiver Operator Curve (ROC) curve was generated which validated the survival analysis of high risk and low risk groups. Area Under Curve (AUC) values showed good results indicating the insignificant False positive rate of the analysis validating the risk model for prediction of OS for 1 year, 3 years, and 5 years (Fig. 5F). Heatmap of the six genes' signature set showed that the hazardous *MDGA2, DLX1,* and *DSC1* genes' expression were higher in the high-risk category whereas the protective genes *GGT2, GNLY*, and *UGT2B4* genes’ expression was higher in the low-risk group (Fig. 5G).

### 3.8 Construction of a nomogram model to improve the predictive power of the risk model

**Fig. 6:**
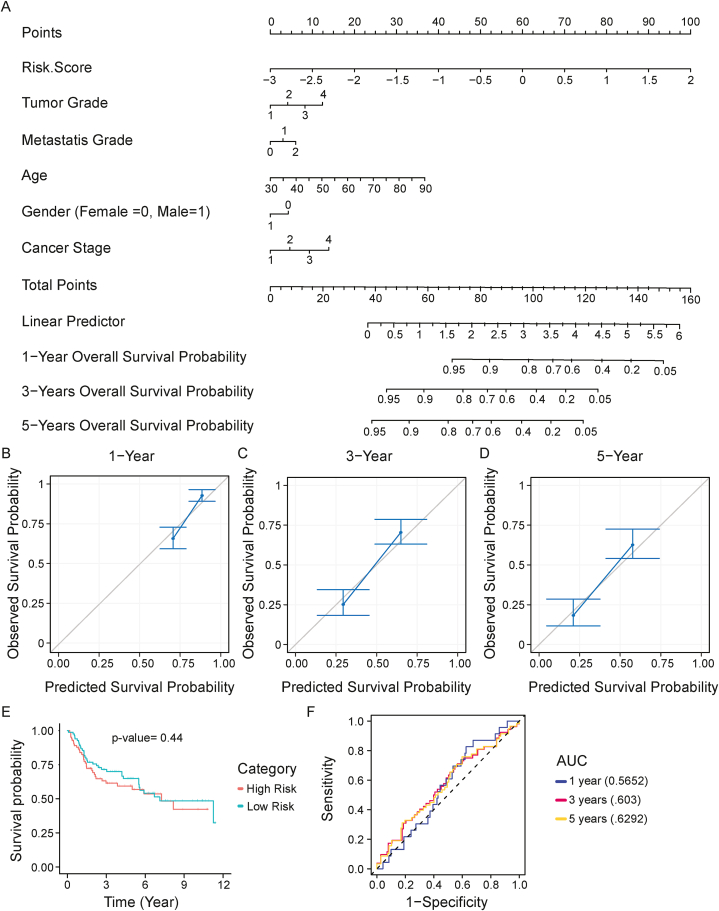
(A) The development of a nomogram including subtype-specific signatures and clinical characteristics for the prediction of survival. **(B**–**D)** Nomogram calibration plot showing actual and predicted probability of the one-, three- and five-years. The result showing actual and prediction values above baseline. **(E**–**F)** Kaplan–Meier survival analysis of GSE13507 (p-value = 0.44), showed no significance. The ROC curve was also generated for the analysis which showed AUC values above the baseline.

A nomogram model was constructed by combining the six-gene signature risk score, and clinical factors including age, gender, metastasis grade, tumor grade, and cancer stage for predicting BLCA patients' overall survival. Nomogram showed consistent results with all the clinical factors. 2 years, 3 years, and 5 years survival probability showed decreasing survival probability with the points (Fig. 6A). The calibration plot showed a prediction value above the baseline for all 3 predictions which cement prediction significance (Fig. 6B–D). Jointly, the nomogram integrating the six-gene signature, grade, age, and gender could enhance the predictive power of BLCA patients’ prognosis.

### 3.9 Evaluation and external validation of the signature model performance

GEO datasets GSE13507 (n = 165) was used as an independent dataset for external validation of risk score and survival analysis. Each sample was assigned a risk score, and the samples were separated into high- and low-risk groups using the best splitting point. Kaplan–Meier analysis of GSE13507 (p-value = 0.44), showed no statistical significance of the difference between the two groups. A ROC curve was also generated for the analysis which showed AUC values above the baseline (Fig. 6E and F).

### 3.10 Expression levels of signature genes in independent BLCA patient cohort

**Fig. 7:**
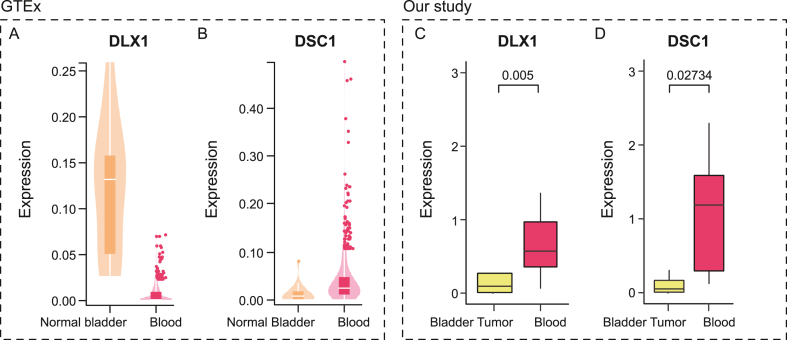
Expression levels of signature genes in BLCA patients of our cohort. **(A-B)** Violin plots illustrating DLX1 and DSC1 gene expression between normal bladder tissue and blood tissue taken from GTEx portal. **(C-D)** Box plot representing the same DLX1 and DSC1 expression levels in the cohort represented in this study.

From GTEx portal, normal bladder tissue and blood tissue expression were analyzed. While *DLX1* gene is upregulated in normal bladder tissues (Fig. 7A), *DSC1* gene showed lower expression (Fig. 7B) compared to blood tissues. The expression of the four target genes (*MDGA2, DLX1, DSC1, and GNLY*) was investigated in tumor tissues and paired blood samples from 10 BLCA patients who were enrolled in the current study. The expression level was very high for both genes in blood tissues compared to the bladder tumor tissues after normalization with the housekeeping gene *ACTB* (Fig. 7C and D). A wilcoxon rank test reveals significant difference between the blood and tumor tissues for *DLX1* (0.005) and *DSC1* (0.02734) genes. Gene expression for *MDGA2* wasn't found in any of the samples, where *GNLY* expression wasn't seen in blood samples.

### 3.11 Stratification of BLCA patients based on the risk prediction

The *project's* long-term objective is to identify high and low risk patient categories in our cohort. Using the same kind of procedure to select two groups from the TCGA-BLCA dataset, a risk score was generated for each sample. From the ten samples that comprised our cohort, the risk formula produced two subtypes. Five samples from each of the groups were divided into low-risk and high-risk categories respectively. The expression levels of the *DLX1*, *DSC1*, and *GNLY* genes were taken into consideration as no *MDGA2* gene expression was found in our cohort. The results are given in Table 1. The demographic and histopathology data summary are given in Supplementary Table 1.

**Table 1:** Gene Expression values and Risk scores of samples of BLCA patients of our cohort.

Tumor Samples	DLX1	DSC1	GNLY	Risk Score	Risk Group
T37	0.395464	0.399748	0.398755	0.14094403	Low Risk
T15	0.409501	0.427576	0.421973	0.14751408	Low Risk
T9	0.42662	0.411521	0.409563	0.15173857	Low Risk
T44	0.423999	0.43461	0.405816	0.1585099	Low Risk
T50	0.47725	0.477138	0.441988	0.17880091	Low Risk
T10	0.417016	0.429026	0.310101	0.17956884	High Risk
T52	0.499292	0.488207	0.458136	0.18501026	High Risk
T39	0.480964	0.475889	0.416388	0.18633474	High Risk
T14	0.483882	0.495983	0.424441	0.19098316	High Risk
T42	0.501269	0.52213	0.442059	0.1996277	High Risk

## 4 Discussion

The aim of this study was to construct a signature gene set serving as a prognostic biomarker for muscle invasive bladder cancer patients. We initially selected hallmark p53 pathway associated genes for prognostic analysis. The univariate analysis generated 45 significant genes that were predicted to be involved with the survival status of the TCGA-BLCA patients [23]. The NMF algorithm generated two distinct clusters of BLCA based on the expression data of these 45 genes. The samples from the two subtypes were subjected to a Kaplan-Meier survival analysis, that showed a difference in survival representing a worse prognosis for C1 subtype patients [25]. Additionally, rather than only exploring specific targeted genes, we analyzed all the genomic regions undergoing recurrent somatic copy-number alterations between the two subtypes generated through the clustering analysis in our study. Interestingly, we observed a significant difference in the frequency of chromosomal amplification or deletion across the C1 and C2 (Supplementary Fig. 2). The C1 group shows a lower frequency of copy number gain and loss in comparison to the C2 subtype. While the common amplification and deletions were common in both subtypes, the difference in the overall frequency rate could explain distinct prognosis in the subtypes [45].

Previously, it was found that cancer-associated proteolytic components of stromal cells are required for tumor invasiveness [46]. We tried to correlate the tumor purity, as well as stromal and immune score results between the previously defined subtypes. Tumor purity is defined by the proportion of cancer cells in tissues [29]. Our previous results revealed that the C1 subtype showed a significantly greater stromal score and immune score than the C2 subtype. To determine the purity of each tumor sample, we employed five different methods- ESTIMATE, ABSOLUTE, LUMP, IHC, and CPE. All the methods specified a uniform result of C1 having a lower purity score than C2.

As an immunocompromised malignancy BLCA tissues are infiltrated by tumor-infiltrating immune cells (TIICs) such as T cells, dendritic cells, macrophages, neutrophils, and mast cells, among others [47]. In previous studies, TIICs, major components of the tumor microenvironment, have been shown to be strongly associated with the efficacy of targeted therapies and clinical outcomes of patients [48]. Implementing the CIBERSORTx algorithm in our sample data resulted in some intriguing outcomes. For example, 17 out of 22 TIICs showed significantly different levels in the subtypes. The proportions of neutrophils, NK cells, macrophages, dendritic cells, and mast cells have been raised or reduced in BLCA as found by previous studies, however, the relationship of CD4 and CD8 cells still remains broadly unknown [17]. Some studies have indicated that CD8 cells contribute to a better prognosis for BLCA, while others have shown that the rationale may be attributed to the combined influence of CD8 and other immune cells [49]. Our analysis revealed that subtype C2 patients with a better prognosis had a greater CD8 cell fraction. However, it is insufficient to conclude that the improved prognosis is due to the CD8 population. Besides, higher CD4 cell counts and CD3/CD4 ratios have been linked to a poor prognosis, which was also confirmed in our investigation for the C1 subtype [50,51]. While BLCA increases the number of NK cells, macrophages, and neutrophils, it decreases the number of dendritic cells, mast cells, and B cells [17,48]. We also discovered the same outcomes in our study's subtypes. While the number of NK cells, macrophages, and neutrophils were greater in the C1 subtype, it was lower in the C2 subtype. The Tregs were lower in the C1 cluster compared to C2 which is interesting as Tregs generally show tumor developmental and immune suppressive role, but there have been cases where results were somewhat different especially for BLCA which can be a case here [52]. Importantly, the immune cell fraction analysis corroborated the prognosis rate in cases of our subtyping and proved it to be pretty accurate.

Next, we investigated the expression of chemokines (*CXCL1, CXCL2, CXCL5 CXCL10, CXCL11, CXCL13*) that have been found associated with BLCA. These factors help induce tumor-associated macrophages (TAMs), and cancer-associated fibroblasts (CAFs)-recruiting myeloid-derived suppressor cells (MDSC), and eventually increase the risk of cancer [[33], [34], [35],^53^]. All these chemokines were found to be expressed highly in the C1 subtype in comparison to C2. Interestingly, a previous study identified *CXCL13* to be associated with a better survival rate, which does not align with our results as the C1 subtype had higher *CXCL13* expression [53]. Immune checkpoint genes *CTLA4, PD1 (CD274), IDO1, LAG3, TIGIT, PDCD1*, and *PDCD1LG2* all exhibited greater expression for the C1 subtype, indicating that the C2 and C1 subtypes have different immunological properties.

Our analysis showed that differentially expressed upregulated C1 subtype genes were highly enriched in immunity-related pathways of KEGG and GO biological process pathway whereby C2 subtype upregulated genes were highly enriched in steroid biosynthesis pathway. Previous works have identified a variety of pathways in bladder cancer that are controlled by steroid hormones and their receptor signals [54]. Nuclear receptor-mediated signals may also have a role in the development of urothelial carcinoma, according to new research [54]. C2 subtype showed higher enrichment in these pathways which might inhibit the progression of the tumor. Estrogen receptor has been seen in reducing cancer development in bladder cell [55]. Besides, Cytochrome P450 (CYP) enzymes are seen to play an important role in the phase I metabolism of medicines and other xenobiotics, as well as catalyzing oxidation and reduction reactions. Intermediates from CYP-dependent metabolism are often harmful or carcinogenic, but they may also be used to create inactive polar products for renal excretion [56]. The C2 subtype exhibited a high enrichment score for cytochrome P450-related pathways, which might be the reason why these patients had a superior drug metabolism response to anticancer treatment as a result of their improved prognosis.

We next implemented a univariate and lasso-based regression model as well as performed a multivariate cox regression analysis on our specified regulated genes that resulted in our six-risk associated genes: *MDGA2*, *DLX1*, *DSC1*, *UGT2B4*, *GNLY*, and *GGT2*. Strikingly, the association of these genes with BLCA has not been established. Both the Lasso regression score and the multivariate analysis regression score indicated that overexpression of *MDGA2*, *DLX1*, and *DSC1* was associated with an increased risk, whereas overexpression of *UGT2B4*, *GNLY*, and *GGT2* were indicative of a protective role against BLCA. Our developed risk score formula (combination of the expression data and the multivariate analysis coefficient score for these six genes) divided the patients into high-risk and low-risk groups. The presence of a significant number of high-risk patients with a higher mortality rate validated the risk model's legitimacy (P = 0.016). We confirmed these findings by conducting a Kaplan-Meier survival analysis between the high- and low-risk groups, which revealed that the high-risk groups had a worse prognosis rate. This result was validated by using a ROC curve analysis, which revealed AUC values above the threshold for 1 year, 3 years, and 5 years-removing any concern about false positive rate values. Besides, we also formed a nomogram model based upon the clinical data and the risk score that provides a numerical likelihood of a clinical occurrence. The analysis revealed the survival probability decreased with time upon the scoring. The calibration plot revealed a satisfactory observed vs projected survival probability score when comparing the two variables. The ratio was higher than the baseline, indicating that the model was effective. Our risk score for six genes was also evaluated using the independent dataset GSE13507. The survival plot didn't provide statistically significant results. The difference in outcome between the 2 sets of data may be explained by the fact that TCGA cohort data was mostly derived from the US population whereas the GSE13507 was from the Korean population. However, we can't conclude anything from this, as data were insignificant and may need more independent dataset(s) to evaluate the results.

Additionally, the gene expression levels of bladder tissue and blood tissue were compared using the GTEx portal (https://gtexportal.org/), and it was discovered that *DLX1* was overexpressed in normal bladder tissue compared to blood tissue, whereas *DSC1* gene expression was lower in normal bladder tissues compared to blood tissues. From the identified signature gene set, *MDGA2, GNLY, DLX1*, and *DSC1* were selected for expression analysis in bladder tumors and matching blood samples of 10 BLCA patients. The qPCR assay for the *MDGA2* gene did not show any CT values for any of the cDNA samples. *GNLY* showed no expression in any of the blood samples, despite the fact that *GNLY* expression was quite high in blood tissues in the GTEx portal. The expression of the other two genes, *DLX1* and *DSC1*, was moderate to high in all of the samples. After using the Livak method [42] and normalizing with the *ACTB* gene, it was found that *DLX1* and *DSC1* expression was lower in tumor tissues than in blood tissues. While *DSC1* gene expression in tumor tissues followed the same pattern as in normal bladder tissues, *DLX1* expression was the reverse. This was intriguing since the families of these two genes have been shown to have both oncogenic and tumor suppressive roles in cancer [[57], [58], [59]]. *DLX* family genes are involved in embryo development. An increased expression of the *DLX1* was seen in prostate cancer confirming the positive regulation of β-catenin signalling [60]. *DSC1* gene produces a desmocollin-subfamily cadherin superfamily calcium-dependent glycoprotein. These desmosomal family members, together with the desmogleins, are sticky proteins of the desmosome cell-cell interface and important for cell adhesion and desmosome formation in epithelial cells [61]. This gene has also been associated with anal carcinoma, head and neck cancer, colorectal cancer, and lung cancer [[62], [63], [64], [65]]. However, the limited size of sample set analysis precludes a conclusive outcome. A risk score was generated for each tumor sample with the expression score of GNLY, DSC1, and DLX1, and patients were categorized into two subtypes as high risk and low-risk groups. Further study will be done in the following years when the survival data will be available and the cohort size is larger.

This research identified a unique signature gene set based on the p53 pathway. Six genes were identified that showed significant association with survival in Bladder Cancer patients. However, this data is not enough to give a conclusive remark on anything. The main limitation of the study is the validation cohort size and lack of validation both with independent dataset and a larger cohort. Another limitation of the study is the lack of survival data in the validation cohort, which is important to the time dependent analysis and confirmation of the risk score formula. We also acknowledge that the relationship between our gene set and TNM status hasn't been thoroughly examined yet. However, it's crucial to highlight that further investigation regarding the TNM status should be done in the future to gain a more comprehensive understanding of the significance of our gene set in clinical context. Currently, the BLCA patients enrolled in this study are in active follow-up for their treatment response and survival. A timely analysis of the association of the expression of these six genes with the survival of BLCA patients is warranted in due time course.

## 5 Conclusions

Our work attempts to identify a predictive characteristic gene set for BLCA. We effectively clustered the TCGA-BLCA datasets into two subtypes using p53 pathway-related genes. We verified the clustering effectively by using different transcriptomics tools and Bioconductor packages. We successfully developed a prognostic signature gene set of six genes (*DLX1, DSC1, MDGA2, UGT2B4, GNLY*, *GGT2*) using differential gene expression, a risk model, and predictive nomogram modeling. Finally, we validated the findings in an independent BLCA cohort and observed a considerable deviation from the expected trend. While *DSC1* had a statistically significant difference in the bladder tumor to blood ratio, *DLX1* had the opposite effect on the bladder tumor to blood ratio. While the sample size is insufficient to draw any conclusions, this might likely become a method for creating signature gene sets.

## Data availability

Data will be made available on request.

## CRediT authorship contribution statement

**Safayat Mahmud Khan:** Data curation, Formal analysis, Investigation, Methodology, Software, Validation, Writing – original draft, Writing – review & editing. **Tonmoy Das:** Data curation, Formal analysis, Investigation, Methodology, Software, Validation, Writing – original draft, Writing – review & editing. **Sajib Chakraborty:** Conceptualization, Data curation, Formal analysis, Funding acquisition, Resources, Software, Supervision, Writing – original draft, Writing – review & editing. **Abdul Matin Anamur Rashid Choudhury:** Project administration, Resources, Validation, Visualization, Writing – original draft, Writing – review & editing. **Howlader Fazlul Karim:** Data curation, Investigation, Resources, Visualization, Writing – original draft, Writing – review & editing. **Munshi Akid Mostofa:** Data curation, Investigation, Methodology, Resources, Supervision, Writing – original draft, Writing – review & editing. **Hasib Uddin Ahmed:** Data curation, Formal analysis, Investigation, Methodology, Validation, Writing – original draft, Writing – review & editing. **Md Akmal Hossain:** Investigation, Methodology, Resources, Validation, Writing – original draft. **Florence Le Calvez-Kelm:** Conceptualization, Data curation, Methodology, Resources, Supervision, Visualization, Writing – original draft, Writing – review & editing. **Md Ismail Hosen:** Conceptualization, Data curation, Formal analysis, Funding acquisition, Investigation, Methodology, Project administration, Resources, Software, Supervision, Validation, Visualization, Writing – original draft, Writing – review & editing. **Hossain Uddin Shekhar:** Conceptualization, Data curation, Formal analysis, Funding acquisition, Methodology, Project administration, Resources, Supervision, Writing – original draft, Writing – review & editing.

## Declaration of competing interest

The authors declare that they have no known competing financial interests or personal relationships that could have appeared to influence the work reported in this paper.
